# Investigation of the Medium- and Long-Term Results of a Pioneering Method in the Treatment of Geriatric Intertrochanteric Femur Fractures: Osteosynthesis Using the WALANT Technique

**DOI:** 10.3390/jcm14176078

**Published:** 2025-08-28

**Authors:** Yusuf Murat Altun, Mete Gedikbaş, Murat Aşçı

**Affiliations:** Department of Orthopedics and Traumatology, School of Medicine, Bilecik Şeyh Edebali University, 11230 Bilecik, Turkey; dr.murataltun@hotmail.com (Y.M.A.); drmgedikbas@gmail.com (M.G.)

**Keywords:** hip fracture, wide-awake local anesthesia (WALANT), local anesthesia, functional outcomes

## Abstract

**Background/Objectives**: Femoral neck and proximal femur fractures in the elderly can result from low-energy trauma due to osteoporotic changes and contribute significantly to increased morbidity and mortality. Despite various treatment options, closed reduction and internal fixation (CRIF) with intramedullary nails has become the predominant approach. While a minimally invasive approach reduces complications and speeds recovery, this outcome is not always feasible in practice. The primary surgical goal remains achieving a stable and precise fracture reduction, favoring CRIF when possible. Our study aims to evaluate the clinical, radiological, and functional outcomes of patients operated on using the Wide-Awake Local Anesthesia No Tourniquet (WALANT) technique. **Methods**: Patients who underwent surgery for intertrochanteric femur fractures between June 2019 and June 2021 were analyzed. Patients who were between 75 and 90 years old and had undergone surgery with a proximal femoral nail (PFN) were included in the study. Patients were excluded if they required general anesthesia, if an acceptable reduction could not be achieved with the PFN, if they did not attend the last follow-up examination, or if the follow-up period was <4 years. Patients were functionally assessed using the Harris hip score at the 6th month and at the last follow-up and using the visual analog scale at the surgery, at the 4th hour after surgery, and at the time of discharge. For radiological assessment, the classification of reduction quality and the measurement of the tip–apex distance were used. **Results**: Forty patients (22F/18M) were included in the study. Their mean age was 83.0 ± 2.9 years. The mean time from trauma to surgery was 6.8 ± 2.3 h. Patients were mobilized on average 1.53 ± 0.8 h after surgery, and the mean hospitalization time was 27.4 ± 8.1 h. No statistically significant decrease in hemoglobin value was observed before or after surgery (*p* = 0.476). The Harris hip score was 73.3 ± 3.2 at the 6th month postoperatively and 74.9 ± 2.5 at the last follow-up (*p* = 0.296). The reduction quality was found to be poor in only two patients. **Conclusions**: The WALANT technique’s promising results in terms of pain management, blood loss control, and early mobilization show that it is a viable alternative to conventional anesthesia methods in geriatric hip fractures.

## 1. Introduction

Hip fractures are associated with morbidity and mortality in elderly patients, particularly those with high frailty [1,2]. As the world population ages, the absolute incidence of hip fractures is expected to increase [3]. In the literature, mortality rates within the first 30 days after surgery range between 6% and 12% [4,5,6,7,8,9,10,11]. Most geriatric hip fractures require surgical intervention [12]. The surgical procedure is usually performed with general or spinal anesthesia.

Numerous morbidities, such as diabetes, heart disease, hypertension, pulmonary disease, and poor general health, are present in this patient group, which is why the recognized standard of care for this type of fracture is to achieve early mobility with a minimally invasive surgical procedure that allows anatomical alignment and stable fixation [13,14]. Biomechanical studies have led to the development of intramedullary implants that are suitable for the treatment of trochanteric fractures due to their load-bearing capacity and good mechanical properties [15].

The Wide-Awake Local Anesthesia No Tourniquet (WALANT) technique owes its name to the fact that it allows the patient to be operated on awake and without a tourniquet during extremity surgery [16]. It offers considerable advantages, such as a hemorrhage-free area, less postoperative pain, faster rehabilitation, and lower costs [17]. Excellent results have been reported for minor surgeries, particularly in the hand, wrist, foot, and ankle [18,19,20,21,22,23,24]. In view of the current information, it appears to be a good alternative to both local anesthesia with a tourniquet and general anesthesia [18,22,24].

As far as we know, no study in the literature has reported on the results of proximal femoral nailing using the WALANT technique. Our study aims to evaluate the functional and radiological outcomes of the patients we operated on with the WALANT technique and to demonstrate this method’s potential advantages compared to other anesthesia methods.

## 2. Materials and Methods

### 2.1. Study Design

Ethical approval for this retrospective study was obtained from the Ethics Committee of the Bilecik Seyh Edebali University School of Medicine (Decision No: 88495, Date: 06.04.2022). All patients provided written informed consent before participating. The study was conducted in accordance with the principles of the Declaration of Helsinki.

This retrospective study examined patients who underwent surgery for pertrochanteric femur fractures between June 2019 and June 2021. The operations were performed at a single center by a single surgeon with 10 years of experience in trauma surgery (Y.M.A.). The preoperative and final follow-up examinations of the patients included in the study were performed by 2 orthopedic and traumatological surgeons who were not involved in the operations (M.A. and M.G.).

This study included patients between 75 and 90 years of age who underwent surgery using a proximal femoral nail (PFN) due to intertrochanteric femur fractures. Exclusion criteria included (1) patients who required general anesthesia during surgery, (2) patients who were not treated with a PFN due to inadequate reduction, (3) patients who did not want to participate in the study, (4) patients who did not attend the final control examination, and (5) patients with a follow-up period of less than 4 years.

### 2.2. Functional and Radiological Outcomes

Preoperatively, the fractures were classified according to the classification of the Arbeitsgemeinschaft für Osteosynthesefragen (AO). At the final follow-up examination, the quality of the reduction was primarily categorized as good, acceptable, or poor using the method proposed by Baumgaertner et al. [25], also taking into account the alignment and displacement of the fragments. For a reduction to be classified as “good,” it had to show normal or slight valgus alignment on the anteroposterior radiograph (less than 20 degrees on a lateral radiograph), and no fragment could be displaced by more than 4 mm. For a reduction to be classified as “acceptable,” it had to fulfill the criteria for a good reduction in terms of both alignment and displacement. A “poor” reduction did not fulfill any of these criteria. The tip–apex distance was the other radiological parameter [25].

The patients’ functional assessment was based on the Harris hip score at the 6th month after surgery and at the last follow-up examination, as well as on the visual analogue scale (VAS) at the 4th hour after surgery and at the time of discharge. Age, gender, time to surgery, pre- and postoperative hemoglobin levels, transfusion requirements, need for analgesics, mobilization time, length of hospital stay, and the occurrence of complications were recorded.

### 2.3. Surgical Technique

**Figure 1:**
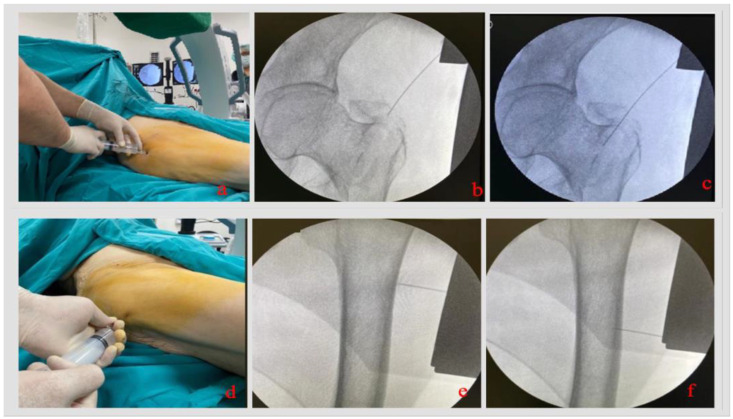
Injection of a local anesthetic under fluoroscopic control. (**a**) The spinal needle is inserted approx. 5 cm proximal to the tip of the greater trochanter. (**b**) The tip of the greater trochanter, where the first access will be made, is reached with the spinal needle under fluoroscopic control. (**c**) The drug is injected into the intramedullary space: The drug is applied into the intramedullary canal by inserting it into the fracture line. (**d**) The distal injection is made proximally. (**e**,**f**) Application of periosteal anesthesia in the estimated area where the lag screw and distal locking screw will be inserted.

Following the patient’s transfer from the transfer stretcher to the surgical table, the anesthesia team controlled vascular access, and additional vascular accesses were opened if anesthesia was deemed necessary. An anesthesiologist and an anesthesia technician monitored the patient for possible side effects throughout the procedure; the patient was observed clinically and communicated with. After putting on sterile gloves, the surgeon who administered the WALANT cleaned the injection site with an iodinated solution and administered the drugs prepared for the anesthesia solution in the required amounts (15 mL of lidocaine, 15 mL of bupivacaine, 2 mL of sodium bicarbonate, and 1 mL of 0.5% adrenaline, made up to 60 ccs with an isotonic solution). Accompanied by fluoroscopy, a spinal needle was inserted into the trochanteric fracture line and the fracture hematoma from the skin entry site for the proximal femoral nail, a few centimeters proximal to the trochanter tip, and the fracture hematoma was confirmed by aspirating the injector (Figure 1). Twenty-five cubic centimeters of the solution was slowly injected into this area; the patient was monitored. After the completion of the hematoma block, the spinal needle was withdrawn slowly, and the injection of 10 cc of the solution was continued until it came out of the skin. Fifteen cubic centimeters of the solution was injected into the periosteum of the estimated insertion site of the femoral neck screws (10 cc), and the injection was continued while the spinal needle was taken out (5 cc). The remaining 10 cc of the solution in the injector was applied to the estimated distal locking screw area of the nail, with puncture injections in the periosteum. Then, the injection was completed while pulling the spinal thread back (at this stage, if the nail size changed or did not coincide with the possible area, a separate application was made for this area; additional local anesthetic might be used). Although the decision was made according to the reduction status of the fracture after traction, if the need to place a hook under the femoral neck was anticipated, an extra 10 cc of the solution might be required in this area. During all injection procedures, control was provided with fluoroscopy. After the injection, we waited 20 min before painting and covering could begin. If the patient had pain while lifting the lower extremity for sterile staining, the total waiting time could reach 30 min until the patient had no pain with the routine orthopedic movements. Before starting the surgical procedure, all injection sites were controlled with forceps to check that anesthesia was provided, and the surgical incision was started as in the standard proximal femoral nailing procedure. Our clinic performs proximal femoral nailing surgery without using a traction table.

### 2.4. Statistical Analysis

Statistical analysis was performed with SPSS 26.0 for Windows (SPSS, Inc., Chicago, IL, USA). The data distribution was evaluated using the Kolmogorov–Smirnov test. The categorical data were assessed using the Pearson chi-square, Fisher’s exact, and Fisher–Freeman–Halton tests. The parametric and nonparametric data were evaluated using Student’s t-test and a Mann–Whitney U test, respectively. For non-normally distributed data, the dependent groups were evaluated using the Shapiro–Wilk and Wilcoxon signed rank tests. A value of *p* < 0.05 was considered significant for all analyses.

## 3. Results

**Figure 2:**
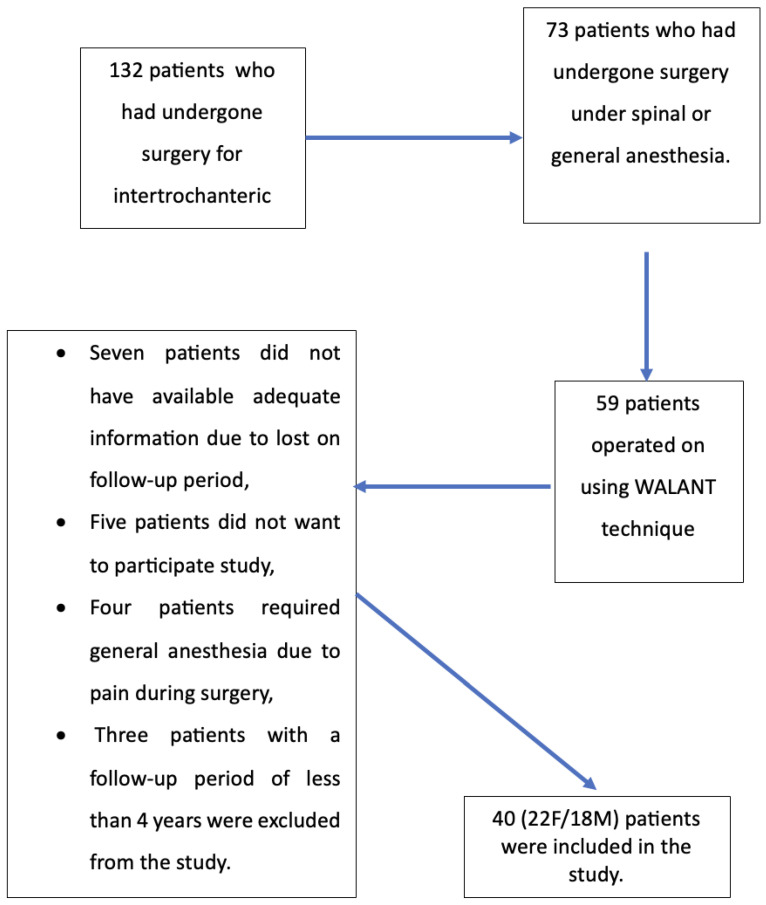
Presentation of the patients participating in our study in the form of a flow chart according to the inclusion and exclusion criteria.

Forty patients (22F/18M) who met the inclusion and exclusion criteria were included in the study (Figure 2). Our patients’ mean age at the time of surgery was 83.0 ± 2.9 years. The mean follow-up time was 52.8 ± 7.2 months. The mean time from trauma to surgery was 6.8 ± 2.3 h. The patients’ descriptive characteristics are listed in Table 1.

**Figure 3:**
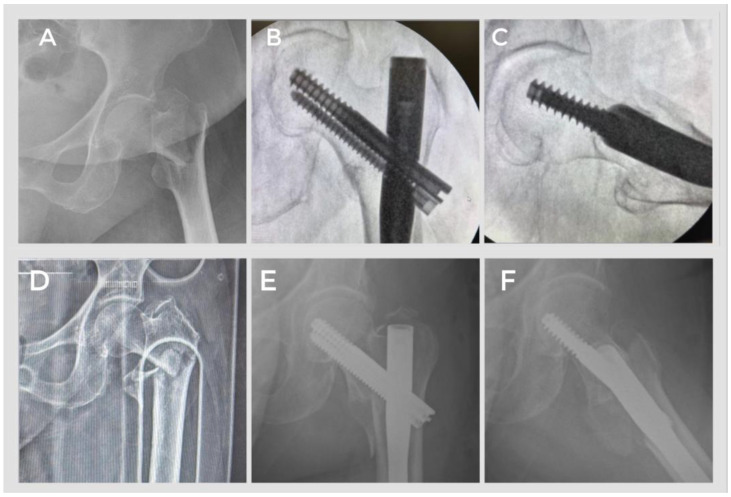
Preoperative and postoperative X-ray images of our patients. (**A**–**C**) A good reduction was achieved in our patient who was operated on for an intertrochanteric femur fracture of type AO 31A1.1. (**D**–**F**) Poor reduction was achieved in our patient who was operated on for an intertrochanteric femur fracture of type AO 31A3.3.

At the last follow-up examination of our patients, the anteroposterior and lateral radiographs taken from the hip radiographs in two directions showed a tip–apex distance of 23.03 ± 0.9 and 23.24 ± 1.2 mm, respectively. When the reduction quality was examined using the method described by Baumgaertner et al., it was found that only 2 patients had poor results; the other 38 patients had acceptable and good results, and union was achieved in all patients. When examining the intraoperative fluoroscopic images of the two patients with poor results, it was found that they had unstable fractures of fracture type 31A3.3, and the reduction phase was difficult (Figure 3).

The patients were mobilized with a walker or crutches after an average of 1.53 ± 0.8 h after the operation. Our patients’ average hospitalization from admission to discharge was 27.4 ± 8.1 h.

When assessing the pain felt during surgery on the VAS, the mean score was 2.53 ± 0.8, while the VAS scores at the 4th hour and at discharge after surgery were 2.6 ± 0.9 and 4.24 ± 1.6, respectively. Our patients’ mean Harris hip score was 73.3 ± 3.2 at the 6th month and 74.9 ± 2.5 at the last follow-up (*p* = 0.288) (Table 2).

While our patients’ preoperative hemoglobin value was 11.2 ± 0.7 g/dL, the hemoglobin value in the 4th hour after the operation was 10.3 ± 1.4 g/dL. No statistically significant decrease was observed before and after surgery (*p* = 0.476). Only one patient required a transfusion of 1 unit of an erythrocyte suspension.

We had a patient who required intensive medical care in the postoperative phase and was discharged from the intensive care unit the day after the operation. After discharge, the hospital records showed that only one patient developed deep vein thrombosis and then pulmonary thromboembolism. Local anesthetic toxicity or other allergic reactions were not observed in any of the patients we operated on.

## 4. Discussion

When we examined our results, we found that early mobilization could be achieved in patients operated on for intertrochanteric femoral fractures using the WALANT technique, that the hemoglobin drop that can result from intraoperative bleeding and the need for blood transfusions were minimal, that patients could be discharged in a very short time, and that both radiological and functional results were quite satisfactory.

One of the most important postulates of orthopedics is early, pain-free mobilization that enables the patient to function again as quickly as possible. The concept of fast-track surgery, which first became popular for elective hip replacement surgery, has shortened the length of hospitalization, reduced complications, and lowered costs [26]. In recent years, research has been carried out on protocols that accelerate the process in trauma patients, including hip fractures [27,28,29]. In our study, we mobilized our patients on average 1.53 ± 0.8 h after the operation. Some patients were able to walk out of the operating theater independently because the application was local and they were awake. In their study, Aprato et al. [28] reported that the mortality rate of patients who could not be mobilized within the first 10 days after surgery was twice as high as that of patients who could be mobilized. In a randomized controlled study, Oldmeadow et al. [29] reported that patients who were ambulatory 1–2 days postoperatively recovered faster, had shorter hospital stays, and needed less assistance after discharge compared to patients who were ambulatory in 3–4 days. Further studies on the WALANT method and our results could open up new avenues in the treatment of hip fractures.

The VAS score is a common method for evaluating intraoperative pain. Mahindra et al. [30] reported that patients’ VAS scores after pericapsular nerve group (PENG) and suprainguinal fascia iliaca compartment blockade (S-FICB) were 3.29 ± 0.73 and 5.1 ± 0.71, respectively. In a study by Tang et al. [31] examining the outcomes of patients who had undergone surgery under general and spinal anesthesia, the patients’ VAS scores were reported as 4 and 5, respectively. Our patients’ VAS scores at the 4th hour after surgery and at discharge were lower than the scores in these studies. According to our pilot study, the WALANT method for intertrochanteric femur fracture surgery was as comfortable and practical as spinal anesthesia and/or combination with the PENG and S-FICB blockade methods.

Pain is a significant issue following intertrochanteric femur fracture surgery. Good pain management after surgery allows for faster rehabilitation, so pain should be treated as soon as possible [32]. In 2018, Giron-Arango et al. [33] defined the method of pericapsular nerve group blockade under ultrasound guidance and used it to reduce pain in five patients with hip fractures. Morrison et al. [34] reviewed the studies presenting cases in which anesthesia was performed using the pericapsular nerve group block method and reported only one study in which surgery was performed using this method in isolation, although the study was withdrawn. In combination with other anesthesia methods, the pericapsular nerve group block reduces pre- and postoperative pain and facilitates rehabilitation [33,34,35,36]. The WALANT technique we described is comparable in its application to the combination of classical hematoma block and pericapsular nerve group block. Like spinal anesthesia, it should be considered as an anesthetic procedure and as a technique for pain control in the postoperative period. It was observed that patients’ pain in the postoperative period was less than in the spinal anesthesia group. However, the technique we demonstrated does not require ultrasound training and can be used under fluoroscopy, which all orthopedic surgeons are used to. It also appears to be just as effective as spinal anesthesia.

A recent study by Tüzün et al. examined the factors affecting blood loss and observed less blood loss in patients who underwent surgery within the first 2 days after trauma [37]. Similarly, a study by Mattisson et al. demonstrated less blood loss and lower transfusion requirements in patients who underwent surgery within the first 24 h [38]. In our study, consistent with the literature, we observed that blood loss and transfusion requirements were quite low. We attribute this result to two key factors: patients underwent surgery within the first 6 h, on average, and the WALANT technique, thanks to the adrenaline it contains, reduces bleeding during incision and dissection without a tourniquet. This may create an additional advantage.

Our study has some limitations. It is a pilot retrospective study and not randomized. The number of patients was limited because the operations were performed by a single surgeon with experience in the WALANT technique, and it is obvious that the findings need to be supported by larger, randomized controlled studies. Furthermore, the effects of concomitant diseases on functional outcomes were not investigated. Cost-effectiveness studies should also be carried out, evaluating the decrease in the need for intensive care and anesthesia costs. However, our study’s strengths are that, in all our patients, both the local anesthesia and the surgery were performed by a single surgeon, using the same amount of solution and the same brand of implants, and that the assessments were performed by two orthopedic surgeons who were not involved in the operations. This pilot study shows that, in addition to the many other methods for which it has already been studied, the WALANT method is also safe and effective in hip fractures.

## 5. Conclusions

This groundbreaking study shows that the WALANT technique is as effective and safe as many other anesthetic techniques, both during and after surgery, in patients undergoing osteosynthesis with PFN for intertrochanteric femoral fractures. It also offers a better cost–benefit ratio due to its shorter hospital stay. However, this method still needs to be supported by prospective, randomized, controlled, and long-term follow-up studies.

## Figures and Tables

**Table 1:** Descriptive characteristics of our patients.

		n	%
Gender	Female	22	55
	Male	18	45
Injured extremity	Right	21	52.5
	Left	19	47.5
Fracture type	31A1	15	37.5
	31A2	17	42.5
	31A3	8	20
Reduction quality	Good	27	67.5
	Acceptable	11	27.5
	Poor	2	5
Comorbid diseases	None	8	20
	Hypertension	32	80
	Diabetes mellitus	13	32.5
	Cardiac disease	12	30
	Chronic lung disease	14	35

**Table 2:** Functional outcomes of the patients.

	Sixth-Month Control	Last Follow-Up	*p*
Harris Hip Score	73.3 ± 3.2	74.9 ± 2.5	0.288
Pain severity	32.2 ± 1.8	33.7 ± 1.8	0.337
Function	34.3 ± 2.1	34.1 ± 1.9	0.449
Absence of deformity	3.1 ± 0.5	3.1 ± 0.5	0.573
Range of motion	3.7 ± 0.8	3.7 ± 0.7	0.573

## Data Availability

Patient data are available from the corresponding author when requested with a reasonable justification.

## Abbreviations

The following abbreviations are used in this manuscript:

WALANT	Wide-awake Local Anesthesia No Tourniquet
PFN	Proximal femoral nail
HHS	Harris hip score
VAS	Visual analog scale
AO	Arbeitsgemeinschaft für Osteosynthesefragen
TAD	Tip–apex distance
AP	Anteroposterior
PENG	Pericapsular nerve group
S-FICB	Suprainguinal fascia iliaca compartment blockade
